# Supplementary material for: Systemic Drivers and Molecular Mechanisms of Sarcopenia in Aetiology‐Specific End‐Stage Liver Disease
Source: J Cachexia Sarcopenia Muscle. 2026 Apr 26;17(3):e70294. doi: 10.1002/jcsm.70294 (PMC13110875; doi:10.1002/jcsm.70294)
Supplement: Supplementary file 1 — Table S1: Demographic data for healthy controls and ESLD patients. Table S2: Sequences of Primers used for qRT‐PCR. Table S3: Demographic data for ESLD subgroups. [file JCSM-17-e70294-s002.docx]

|  | Healthy Control | ESLD | P-value |
| --- | --- | --- | --- |
| Demographics |  |  |  |
| n | 18 | 40 | - |
| Male/Female | 11/7 | 24/16 | - |
| Age (years) | 49.7 ± 15.0 | 54.18 ± 10.66 | 0.2 |
| Height (m) | 1.70 ± 0.1 (*n=*17) | 1.71.± 0.13 | 0.4 |
| Current smoker (n, %) | 1 (6.6) *(n=15)* | 2 (5) | - |
| Ex-smoker (n, %) | 2 (13.3) *(n=15)* | 17 (43) | - |
| Current alcohol consumption (Units/week) | 2 (5) *(n=15)* | (0,0) | **-** |
| Co-Morbidities (n, %) |  |  |  |
| Cardiovascular disease | 0 | 4 | **-** |
| Chronic kidney disease | 0 | 8 | **-** |
| Chronic obstructive airway disease | 0 | 5 | **-** |
| Diabetes mellitus | 0 | 11 | **-** |
| Hypercholesterolaemia | 0 | 6 | **-** |
| Hypertension | 0 | 12 | **-** |
| Insulin dependent diabetes mellitus | 0 | 9 | **-** |
| Metabolic Profile |  |  |  |
| BMI (kg/m^2^) | 24.6 ± 3.5 *(n=17)* | 30.0 ± 6.6 | **0.001** |
| Body fat (%) | 27.5 ± 7.7 (*n=*16) | 30.4 ± 10.6 | 0.3 |
| Body fat mass (kg) | 19.9 ± 6.4 (*n=*16) | 28.7 ±14.1 | **0.04** |
| Dry BMI (kg/m^2^) | 24.4 (5.1) (*n=17*) | 27.4 (9.0) (*n=31)* | **0.014** |
| Dry weight (kg) | 73.0 ± 12.3 (*n=17*) | 85.5 ± 19.3 (*n=31)* | **0.01** |
| Fat free mass (kg) | 51.0 ± 11.5 (*n=*16) | 61.8 ± 12.7 | **0.005** |
| HbA1c (mmol/mol) | 33.8 ± 4.3 | 38.0 ± 15.7 | **0.8** |
| Quad IMAT (%) | 5.2 ± 1.8 *(n=17)* | 10.6 ± 3.6 *(n=38)* | **<0.0001** |
| Waist: Hip | 0.8 ± 0.06 | 1.0 ± 0.09 *(n=37)* | **<0.0001** |
| Weight (kg) | 73.0 ± 12.3 (*n=*17) | 90.6 ± 22.0 | **0.003** |
| Skeletal muscle structure and function |  |  |  |
| Maximal dominant hand grip strength (Kg) | 36.9 ± 10.5 | 31.6 ± 8.5 (*n=38)* | 0.05 |
| Peak knee extensor toque (Nm) | 142.5 ± 51.1 | 101.9 ± 37.4 | **0.0012** |
| Peak quad ACSA (cm^2^) | 64.8 ± 17.8 | 53.8 ± 12.2 (*n=34*) | **0.02** |
| Overall daily activity (mg) | 29.2 ± 8.9 *(n=17)* | 19.2 ± 17.4 *(n=31)* | **0.0001** |
| Quad volume (cm^3^) | 1227.3 ± 393.5 | 1050.0 ± 270.0 (*n=34*) | 0.05 |
| VL muscle thickness (cm) | 2.4 ± 0.5 | 2.1 ± 0.4 | **0.03** |
| LFI | 2.8 ± 0.6 | 3.6 ± 0.5 | **0.0001** |
| Disease Type (n, %) |  |  |  |
| ArLD | - | 18 (45) | - |
| IALD | - | 16 (40) | - |
| MASLD | - | 6 (15) | - |
| ESLD Severity |  |  |  |
| Childs-Pugh Score | - | 7.6 ± 1.7 | - |
| MELD | - | 13.5 ± 4.7 | - |
| UKELD | - | 52.7 ± 3.2 | - |
| Complications of ESLD (n, %) |  |  |  |
| Ascites | - | 29 (73) | - |
| Large volume paracentesis | - | 8 (20) | - |
| Encephalopathy | - | 22 (55) | - |
| Portal hypertension | - | 36 (90) | - |
| Portal vein thrombosis | - | 4 (10) | - |
| Spontaneous bacterial peritonitis | - | 11 (28) | - |
| TIPS | - | 1 (3) | - |
| Blood Analysis |  |  |  |
| Albumin | 42.7 ± 2.6 | 35.2 ± 6.0 | **0.0007** |
| ALP | 71.5 (18.3) | 165.0 (196.5) | **<0.0001** |
| ALT | 18 (6) | 29.5 (36.0) | **0.004** |
| Bilirubin | 12.0 (8.0) | 34.5 (30.0) | **<0.0001** |
| Creatinine | 81 (20) | 73.5.0 (28.5) | 0.5 |
| INR | 1.0 (0.1) (*n=17)* | 1.2 (0.3) | **<0.0001** |
| Platelets | 237 (61.3) | 91.5 (57.5) | **<0.0001** |
| Potassium | 4.2 (0.3) | 4.3 (0.5) | 0.3 |
| Sodium | 139.9 ± 1.8 | 137.2 ± 3.6 | **0.004** |
| Urea | 5.1 (1.2) | 5.9 (4.9) | 0.2 |
| WCC | 4.9 (0.7) | 4.2(3.1) | 0.1 |

**Supplementary Table 1. Demographic data for healthy controls and ESLD patients**

Parametric data are reported as mean ± SD, non-parametric data are reported as median (IQR). Statistical significance was determined by either unpaired Student's *t*-test or Mann–Whitney test for parametric and non-parametric data respectively. Dry bodyweight/BMI was determined by subtracting the percentage of fluid based on clinical examination for mild (5%), moderate (10%), and severe (15%) ascites and/or mild (5%) and moderate (10%) peripheral oedema. Measures of quadricep muscle mass relate to the dominant leg, maximal knee extensor force was measured in the non-dominant leg. ACSA, anatomical cross-sectional area; ALP, alkaline phosphatase; ALT, alanine aminotransferase; INR, International normalised ratio; LFI, liver frailty index; MELD, model for end-stage liver disease; TIPPS, trans-jugular intrahepatic porto-systemic shunt; UKELD, UK model for end-stage liver disease; WCC, white cell count; VL, vastus lateralis.


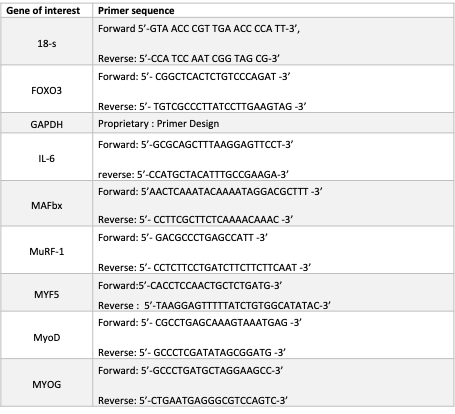


Supplementary Table 2: Sequences of Primers used for qRT-PCR.

|  | ArLD | P-value | MASLD | P-value | Immune Mediated | P-value |
| --- | --- | --- | --- | --- | --- | --- |
| Demographics |  |  |  |  |  |  |
| n | 18 | - | 6 | - | 16 | - |
| Male/Female | 11/7 |  | 2/4 |  | 11/5 | - |
| Age (years) | 55.9±5.0 | 0.3 | 49.0±8.1 | 0.9 | 54.1±15.0 | 5.1 |
| Height (m) | 1.7±0.1 | 0.8 | 1.7±0.1 | 0.4 | 1.8±0.1 | 0.5 |
| Current smoker (n, %) | 1 (6) | - | 0 (0) | - | 1 (6) | - |
| Ex-smoker (n, %) | 10 (56) | - | 3 (50) | - | 4 (25) | - |
| Current alcohol consumption (Units/week) | 0 | - | 0 | - | 0 | - |
| Co-Morbidities (n, %) |  |  |  |  |  |  |
| Cardiovascular disease | 2 (11) | - | 2 (33) | - | 0 (0) | - |
| Chronic kidney disease | 5 (27) | - | 3 (50) | - | 0 (0) | - |
| Chronic obstructive airway disease | 2 (11) | - | 1 (17) | - | 3 (19) | - |
| Diabetes mellitus | 5 (27) | - | 3 (50) | - | 2 (13) | - |
| Hypercholesterolaemia | 1 (6) | - | 2 (33) | - | 3 (19) | - |
| Hypertension | 3 (17) | - | 5 (83) | - | 3 (19) | - |
| Insulin dependent diabetes mellitus | 5 (27) | - | 3 (50) | - | 1 (6) | - |
| Metabolic Profile |  |  |  |  |  |  |
| BMI (kg/m^2^) | 31.8±5.8 | **0.001** | 34.4±7.4 | **0.007** | 27.1±5.4 | 0.7 |
| Body fat (%) | 33.2±8.1 | 0.2 | 35.5±12.6 | 0.2 | 24.0±10.7 | 0.6 |
| Body fat mass (kg) | 32.0±12.2 | **0.01** | 36.4±16.5 | **0.005** | 19.9±11.7 | 1 |
| Dry BMI (kg/m^2^) | 28.4±5.0 | 0.08 | 30.1±6.2 | 0.08 | 28.0±5.8 | 0.09 |
| Dry weight (kg) | 85.8±16.2 | 0.1 | 86.6±27.1 | 0.1 | 87.0±20.5 | 0.1 |
| Fat free mass (kg) | 64.8±14.2 | **0.01** | 61.9±12.6 | 0.16 | 59.2±12.3 | 0.2 |
| HbA1c (mmol/mol) | 33.0 (26.5-52.3) | 1 | 51.0 (26.0-76.5) | 1 | 33.0 (27.3-35.8) | 0.8 |
| Quad IMAT (%) | 11.4 (9.8-13.6) | **<0.0001** | 10.0 (7.3-14.1) | **0.01** | 9.7 (6.8-14.0) | **0.001** |
| Waist: Hip | 1.0±0.1 | **<0.0001** | 1.0±0.1 | **0.006** | 0.9±0.1 | 0.06 |
| Weight (kg) | 95.7±0.7 | **0.004** | 90.7±26.8 | **0.002** | 83.2±20.5 | 0.1 |
| Skeletal muscle structure and function |  |  |  |  |  |  |
| Maximal dominant hand grip strength (Kg) | 32.5±7.7 | 0.39 | 24.9±8.3 | **0.02** | 33.3±8.9 | 0.6 |
| Peak knee extensor toque (Nm) | 96.0±34.0 | **0.01** | 103.2±52.5 | 0.9 | 108.3±39.3 | 0.8 |
| Peak quad ACSA (cm^2^) | 54.9±10.9 | 0.2 | 55.6±14.2 | 0.6 | 55.2±13.2 | 0.3 |
| Overall daily activity (mg) | 22.6±9.7 (n=10) | **0.05** | 13.9 *(n=1)* | - | 17.0±5.6 *(n=10)* | **0.0008** |
| Quad volume (cm^3^) | 1107.0 (983.0-1244.0) | 0.9 | 1078.0 (803.0-1316.0) | 0.9 | 986.0 (909.0-1238.0) | 0.9 |
| VL muscle thickness (cm) | 2.3±0.3 | 0.7 | 2.2±0.5 | 0.6 | 2.1±0.4 | 0.08 |
| LFI | 3.7±0.6 | **<0.0001** | 3.8±0.2 | **0.001** | 3.4±0.5 | **0.003** |
| ESLD Severity |  |  |  |  |  |  |
| Childs-Pugh Score | 8.8±1.2 | - | 7.2±1.5 | - | 6.5±1.5 | - |
| MELD | 14.2±5.0 | - | 13.8±6.1 | - | 12.0±3.0 | - |
| UKELD | 54.0±3.6 | - | 52.6±3.3 | - | 51.5±2.2 | - |
| Complications of ESLD (n, %) |  |  |  |  |  |  |
| Ascites | 17 (95) | - | 4 (67) | - | 7 (44) | - |
| Large volume paracentesis | 5 (28) | - | 1 (17) | - | 1 (6) | - |
| Encephalopathy | 14 (78) | - | 4 (67) | - | 4 (25) | - |
| Portal hypertension | 17 (95) | - | 6 (100) | - | 12 (75) | - |
| Portal vein thrombosis | 5 (28) | - | 0 (0) | - | 0 (0) | - |
| Spontaneous bacterial peritonitis | 7 (39) | - | 1 (17) | - | 1 (6) | - |
| TIPSS | 0 (0) | - | 0 (0) | - | 1 (6) | - |
| Blood Analysis |  |  |  |  |  |  |
| Albumin | 34.1±6.0 | **< 0.0001** | 35.3±6.5 | **0.004** | 34.9±5.9 | **0.0001** |
| ALP | 122.2 ± 39.1 | **0.006** | 124.7±49.8 | 0.08 | 347.7±191.5 | **< 0.001** |
| ALT | 20.0 (16.0-30.0) | 1 | 26.5 (18.3-33.0) | 0.84 | 64.0 (40.0-109.0) | **< 0.0001** |
| Bilirubin | 34.5 (20.5-74.3) | **< 0.0001** | 23.5 (16.3-52.5) | **0.05** | 35.0 (23-50) | **<0.0001** |
| Creatinine | 72.0 (55.0-56.0) | 1 | 70.5 (66.5-145.8) | 1 | 76.0 (58.0-94.0) | 1 |
| INR | 1.3±0.2 | **0.0005** | 1.4 ± 0.3 | **0.05** | 1.3 ± 0.6 | 0.3 |
| Platelets | 86.0 (112.0 – 64.0) | **<0.0001** | 88.5 (62.5-114.3) | **0.0026** | 96.0 (54.0-235.0) | **0.0022** |
| Potassium | 4.3 (4.6-3.9) | 1 | 4.4 (4.1-4.5) | 0.6 | 4.2 (4.1-4.6) | 1 |
| Sodium | 136.0 (135.0-140.0) | **0.02** | 137.0 (132.0-139.0) | 0.09 | 137.5 (137.0-141.0) | 0.8 |
| Urea | 6.4 (3.9-9.7) | **0.0012** | 8.0 (3.2-13.5) | **0.007** | 5.0 (4.2-7.2) | 0.7 |
| WCC | 3.8 (3.3-4.5) | **0.05** | 4.8 (3.7-6.6) | 1 | 5.5 (3.6-6.7) | 1 |

**Supplementary Table 3: Demographic data for ESLD Subsgroups.**

Parametric data are reported as mean ± SD, non-parametric data are reported as median (IQR). Statistical significance was determined by either one-way ANOVA followed by Dunnett’s multiple comparison tests or Kruskal-Wallace tests followed by Dunn’s multiple comparison tests, compared to healthy controls, for parametric and non-parametric data respectively. P values reported are the results of post-hoc tests vs the health control group. Dry bodyweight/BMI was determined by subtracting the percentage of fluid based on clinical examination for mild (5%), moderate (10%), and severe (15%) ascites and/or mild (5%) and moderate (10%) peripheral oedema. Measures of quadricep muscle mass relate to the dominant leg, maximal knee extensor force was measured in the non-dominant leg. ACSA, anatomical cross-sectional area; ALP, alkaline phosphatase; ALT, alanine aminotransferase; INR, International normalised ratio; LFI, liver frailty index; MELD, model for end-stage liver disease; TIPPS, trans-jugular intrahepatic porto-systemic shunt; UKELD, UK model for end-stage liver disease; WCC, white cell count; VL, vastus lateralis.
