# Supplementary material for: Systemic Drivers and Molecular Mechanisms of Sarcopenia in Aetiology‐Specific End‐Stage Liver Disease
Source: J Cachexia Sarcopenia Muscle. 2026 Apr 26;17(3):e70294. doi: 10.1002/jcsm.70294 (PMC13110875; doi:10.1002/jcsm.70294)
Supplement: Supplementary file 2 — Figure S1: MYOG, MyoD and MYF5 mRNA expression in primary human myoblasts/myotubes determined by qRT‐PCR, following stimulation with or without recombinant human HGF (A–C) or GDF‐15 (D–F) for 2, 4 or 8 days. n = 3 biological replicates per condition except D4 and D8 untreated controls where n = 6. *Denotes p value < 0.05. Figure S2: Secretion of IL‐6 protein, from primary human myotubes following 4‐h stimulation with IL‐1, measured by ELISA. Culture media was switched to serum free media upon starting cytokine stimulations, to avoid measurement of IL‐6 derived from FBS. n = 3 patient replicates. Figure S3: mRNA expression of atrophy associated genes; MAFbx, MuRF1 and FOXO3 and IL‐6 in primary human myotubes following stimulation with or without recombinant human HGF (A–D) or GDF‐15 (E‐H) for 24 h at the end of the differentiation period. n = 4 biological replicates per condition. Figure S4: GFRAL mRNA expression in primary human myotubes (n = 4 patient replicates) and undifferentiated and differentiated neurones. [file JCSM-17-e70294-s001.pdf]

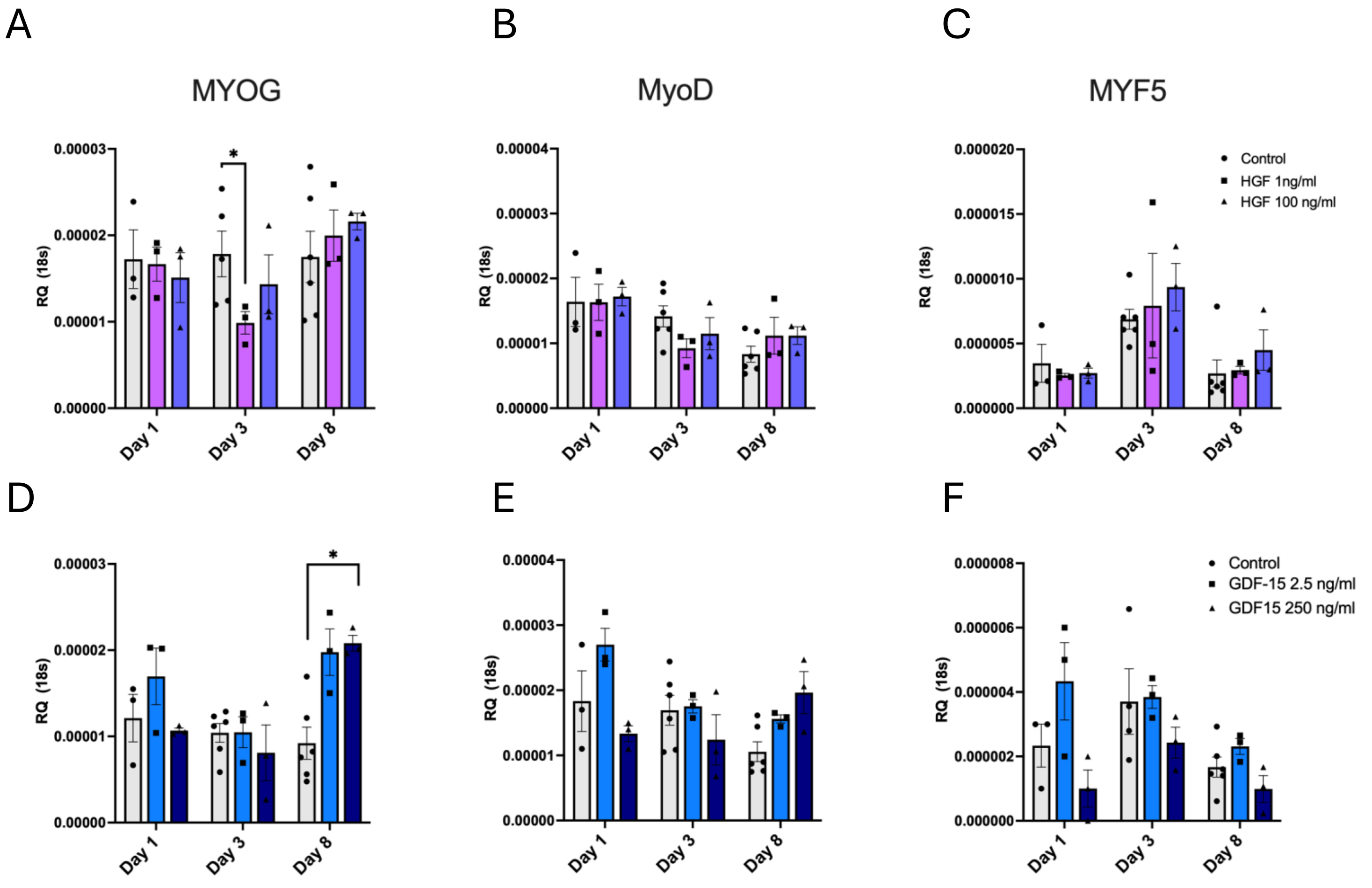

Supplementary Figure 1

MYOG, MyoD and MYF5 mRNA expression in primary human myoblasts/ myotubes determined by qRT-PCR, following stimulation with or without recombinant human HGF (A-C), or GDF-15 (D-F) for 2, 4 or 8 days. n=3 biological replicates per condition except D4 and D8 untreated controls where n=6. * Denotes P value < 0.05).

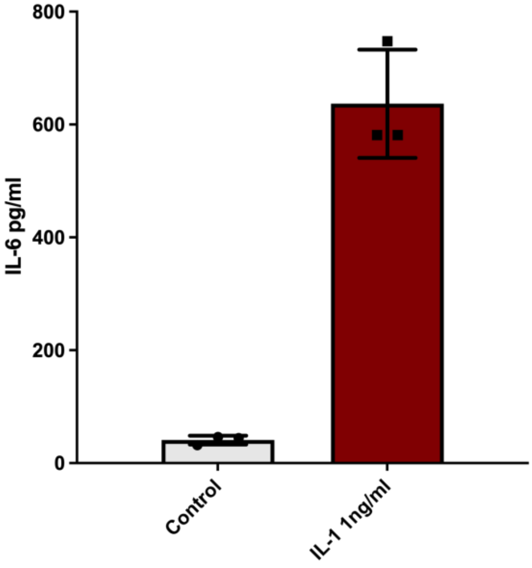

Supplementary Figure 2

Secretion of IL-6 protein, from primary human myotubes following 4h stimulation with IL-1, measured by ELISA. Culture media was switched to serum free media upon starting cytokine stimulations, to avoid measurement of IL-6 derived from FBS. N=3 patient replicates.

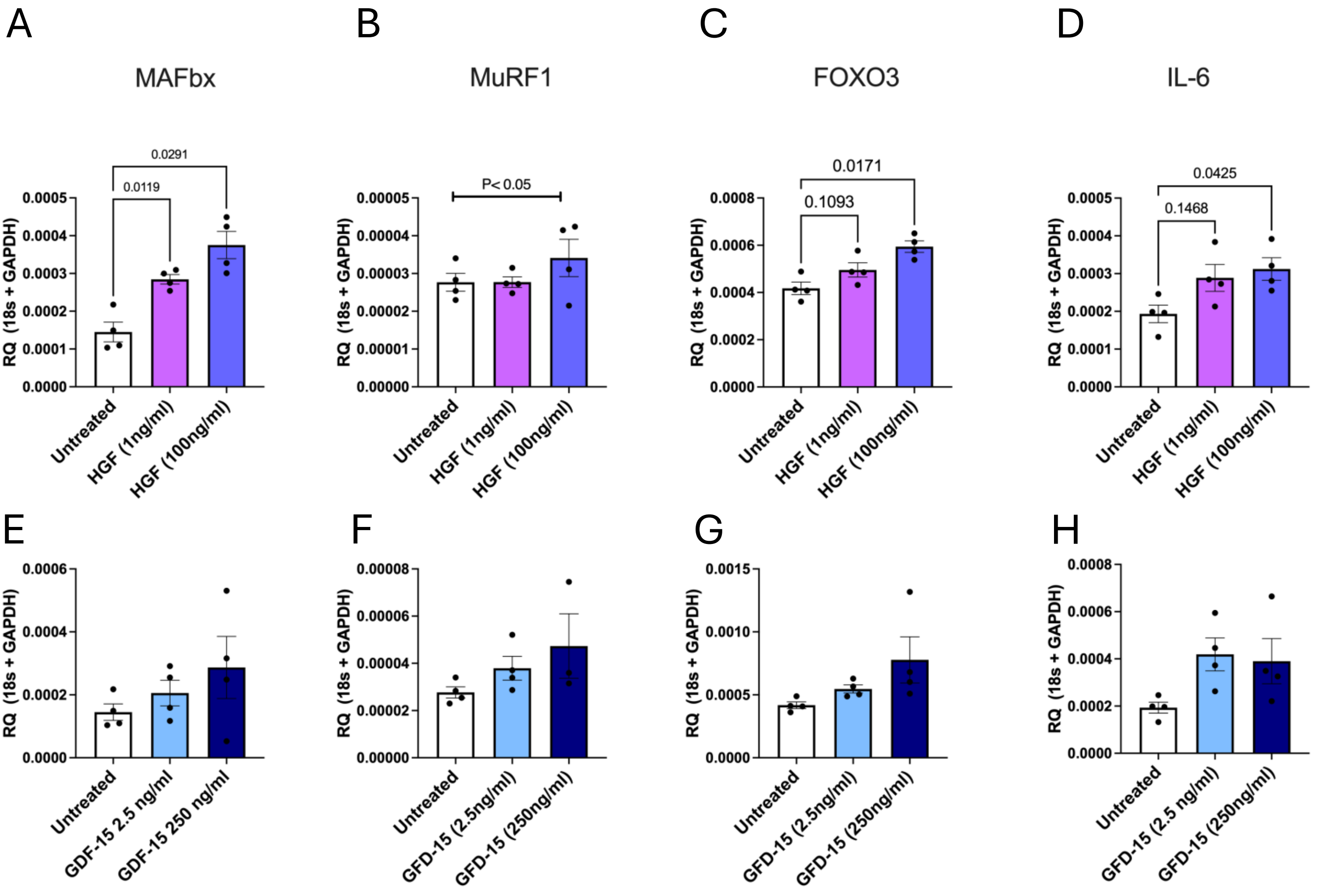

Supplementary Figure 3

mRNA expression of atrophy associated genes; MAFbx, MuRF1 and FOXO3 and IL-6 in primary human myotubes following stimulation with or without recombinant human HGF (A-D) or GDF-15 (E-H) for 24 h at the end of the differentiation period. n=4 biological replicates per condition.

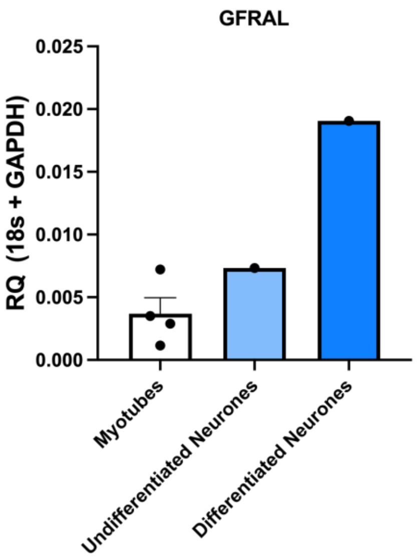

Supplementary Figure 4

GFRAL mRNA expression in primary human myotubes (n=4 patient replicates) and undifferentiated and differentiated neurones.
